# Identification of subphenotypes in critically ill thrombocytopenic patients with different responses to therapeutic interventions: a retrospective study

**DOI:** 10.3389/fmed.2023.1166896

**Published:** 2023-04-27

**Authors:** Xuandong Jiang, Weimin Zhang, Yuting Pan, Xuping Cheng

**Affiliations:** Intensive Care Unit, Dongyang Hospital of Wenzhou Medical University, Jinhua, Zhejiang Province, China

**Keywords:** thrombocytopenia, subphenotypes, fluid resuscitation, artificial intelligence, latent profile analysis, critically ill

## Abstract

**Introduction:**

The causes of thrombocytopenia (TP) in critically ill patients are numerous and heterogeneous. Currently, subphenotype identification is a popular approach to address this problem. Therefore, this study aimed to identify subphenotypes that respond differently to therapeutic interventions in patients with TP using routine clinical data and to improve individualized management of TP.

**Methods:**

This retrospective study included patients with TP admitted to the intensive care unit (ICU) of Dongyang People’s Hospital during 2010–2020. Subphenotypes were identified using latent profile analysis of 15 clinical variables. The Kaplan–Meier method was used to assess the risk of 30-day mortality for different subphenotypes. Multifactorial Cox regression analysis was used to analyze the relationship between therapeutic interventions and in-hospital mortality for different subphenotypes.

**Results:**

This study included a total of 1,666 participants. Four subphenotypes were identified by latent profile analysis, with subphenotype 1 being the most abundant and having a low mortality rate. Subphenotype 2 was characterized by respiratory dysfunction, subphenotype 3 by renal insufficiency, and subphenotype 4 by shock-like features. Kaplan–Meier analysis revealed that the four subphenotypes had different in-30-day mortality rates. The multivariate Cox regression analysis indicated a significant interaction between platelet transfusion and subphenotype, with more platelet transfusion associated with a decreased risk of in-hospital mortality in subphenotype 3 [hazard ratio (HR): 0.66, 95% confidence interval (CI): 0.46–0.94]. In addition, there was a significant interaction between fluid intake and subphenotype, with a higher fluid intake being associated with a decreased risk of in-hospital mortality for subphenotype 3 (HR: 0.94, 95% CI: 0.89–0.99 per 1 l increase in fluid intake) and an increased risk of in-hospital mortality for high fluid intake in subphenotypes 1 (HR: 1.10, 95% CI: 1.03–1.18 per 1 l increase in fluid intake) and 2 (HR: 1.19, 95% CI: 1.08–1.32 per 1 l increase in fluid intake).

**Conclusion:**

Four subphenotypes of TP in critically ill patients with different clinical characteristics and outcomes and differential responses to therapeutic interventions were identified using routine clinical data. These findings can help improve the identification of different subphenotypes in patients with TP for better individualized treatment of patients in the ICU.

## 1. Introduction

Thrombocytopenia (TP) is generally defined as having a platelet count of <100 × 109/L. This condition is common among critically ill patients in both medical and surgical intensive care units (ICUs), with a global prevalence of 21–77% (1, 2). The causes of TP in ICU patients vary, including sepsis, trauma, surgery, and medication (2, 3). Most patients develop TP within 4 days of admission to the ICU. A long duration of TP is associated with a poor prognosis (4, 5). Numerous studies have demonstrated that TP is an independent risk factor for mortality in ICU patients, being associated with severe bleeding events and increased transfusion requirements as well as with the duration of ICU stay and an increased incidence of acute kidney injury (AKI) (6, 7). Unfortunately, the efficacy of current interventions and treatment methods for TP in ICU patients is limited (8, 9).

Previous studies based on standardized treatment regimens for patients with TP have failed to yield satisfactory treatment outcomes. For example, a meta-analysis of the therapeutic efficacy of recombinant human thrombopoietin in patients with TP with sepsis by Zhang et al. revealed no significant difference in 28-day mortality (10). A recent review reported that the use of platelet transfusion, glucocorticoids, and intravenous immune globulin for the treatment of immune TP requires further study (11). The possible reasons for the unsatisfactory treatment outcomes in patients with TP include the significant heterogeneity of TP, which is associated with the presence of multiple pathogenic factors, such as inflammation, endothelial dysfunction, coagulopathy, hemodilution, and altered platelet production, in critically ill patients (12). Subphenotyping, a precision medicine-based treatment option, is currently a very common approach for addressing disease heterogeneity and has been applied to common critical illnesses, such as sepsis, AKI, and acute respiratory distress syndrome (ARDS) (13–15).

Most studies have focused on determining prognosis by staging, and only few studies have focused on different responses to treatment after staging. For example, Zhang et al. retrospectively analyzed 14,993 patients with severe sepsis and identified four subphenotypes of sepsis using latent profile analysis, each of which responded differently to fluid resuscitation (16). Bhatraju et al. used latent class analysis to classify a critically ill AKI population and applied it to AKI patients in the Vasopressin and Septic Shock Trial. The result of the initial analysis was negative, but subphenotyping revealed that vasopressin therapy had survival benefits in patients with subphenotype 1 (17). However, only few studies have reported on the subphenotypes of severe TP, and even fewer studies have reported on its response to different therapeutic interventions (12).

Therefore, this study aimed to identify different subphenotypes in TP patients admitted to the ICU of our hospital over the last 10 years with different clinical outcomes and different responses to therapeutic interventions, using latent profile analysis based on routine clinical data, with the aim of improving prognosis prediction and treatment of critically ill patients and providing guidance for clinicians to achieve individualized management of patients.

## 2. Materials and methods

### 2.1. Study design

This study followed the Strengthening the Reporting of Observational Studies in Epidemiology guidelines (Supplementary Table S1). In this retrospective study, 1,666 patients with TP who were first admitted to the ICU of Dongyang People’s Hospital between January 1, 2010, and October 31, 2020, were included. The inclusion criteria were first admission to the ICU and ICU stay of ≥48 h. The exclusion criteria were age < 18 years, hematological malignancy, liver cirrhosis, or previous splenectomy.

### 2.2. Data collection and grouping

#### 2.2.1. Data collection

Data were collected using the medical record information mining software provided by Shanghai Le9 Healthcare Technology Co., Ltd. (Shanghai, China). The following information was collected: (1) age, sex, Acute Physiology and Chronic Health Evaluation (APACHE)-II score, complications, vasopressor use, renal replacement therapy, fluid intake and urine output for 24 h after ICU admission; and biochemical indexes and first vital signs at ICU admission.

The therapeutic interventions include glucocorticoid use, immunoglobulin use, platelet transfusion during ICU stay, and fluid intake for 24 h after ICU admission.

The primary outcome was hospital mortality. The secondary outcomes included duration of mechanical ventilation, length of ICU stay, length of hospital stay, and hospitalization cost.

#### 2.2.2. Diagnostic criteria

We defined TP as a platelet count of <100 × 10^9^/L in the first 48 h after ICU admission (2, 3).

### 2.3. Data processing

Variables with >20% missing values were deleted. If the incidence of missing values was <2%, the mean value of the variable was substituted for the missing values. The missing values of variables with loss rates of >2 and < 15% were replaced using multiple imputations. Outliers were handled as missing values.

### 2.4. Latent profile analysis

Latent profile analysis (LPA), an unsupervised machine learning algorithm, is a modeling approach for classifying latent variables that focuses on identifying potential subgroups within a population, based on a specific set of variables, using an expectation–maximization algorithm to estimate the parameters of the latent class model (18). The variables included in LPA modeling are clinical and are incorporated from domain expertise and from the relevant literature (16, 19, 20). Pearson’s correlation analysis was used to determine the correlations among characteristic variables, and variables with correlation coefficients >0.7 were removed. Finally, the following 15 common clinical variables were selected: platelet count at initial admission to ICU, age, creatinine level, glucose concentration, systolic blood pressure, respiratory rate, oxygen saturation, heart rate, white blood cell count, hematocrit level, lactate level, pH, partial pressure of oxygen, partial pressure of carbon dioxide, and bicarbonate level. The number of categories was determined using the Bayesian information criterion (BIC), entropy, and bootstrap likelihood ratio tests. Lower BIC values indicated a better model fit. Entropy ranged from 0 to 1, with higher values indicating higher accuracy of categorization. The Vuong–Lo–Mendell–Rubin likelihood ratio test (LRT) was used to assess the number of mixture components in a given finite mixture model parameterization, and value of ps were reported to compare *n*-class and (*n* − 1)-class models (21). A value of *p* of <0.05 indicated statistical significance in the LRT. In addition, the proportion of patients in each potential class with a number of patients of >5% of any other potential class should be assigned to a class with a minimum probability greater than 0.8, otherwise members of this class were considered unstable (22). The number of potential classes was determined in conjunction with clinical interpretation.

### 2.5. Statistical analyses

Descriptive statistics were analyzed conventionally using the CBCgrps package in R^1^ (23). Normally distributed measurement data are expressed as mean and standard deviation (*x* ± *s*), and non-normally distributed data are expressed as median [interquartile range (IQR): P25, P75]. Comparisons across groups on baseline characteristics were performed using analysis of variance for continuous variables and the chi-square tests for categorical variables. All statistical analyses were performed using R (software version 4.1.3; https://www.r-project.org/). A value of *p* <0.05 was considered statistically significant.

The Kaplan–Meier method was used to analyze the relationships of the four subphenotypes with in-hospital 30-day mortality. Multivariate Cox regression models were used to investigate the independent association between therapeutic interventions and mortality. Variables with *p* < 0.1 in the univariate regression analysis and the important clinical variables were selected for the Cox model to test for interactions between different categories and therapeutic interventions. The model was adjusted for the following covariates: age, sex, APACHE II score, vasopressor used, surgery, sepsis and white blood cell count. Platelet transfusion and fluid intake separately interacted with each category. The hazard ratio (HR) and associated 95% confidence interval (CI) for the effect of platelet transfusion and each 1 l increase in fluid intake on mortality outcomes are reported.

### 2.6. Ethics approval

This study was approved by the Ethics Committee of Dongyang People’s Hospital (DRY-2023-YX-016) and followed all related local guidelines and regulations, including the human genetics-related regulations. The need for obtaining informed consent was waived by the Ethical Committee of Dongyang People’s Hospital, due to the retrospective nature of this study, and the study involved no human tissue collection and storage process. The data were analyzed anonymously by removing personal information of the patients.

## 3. Results

### 3.1. Study population

**Figure 1:**
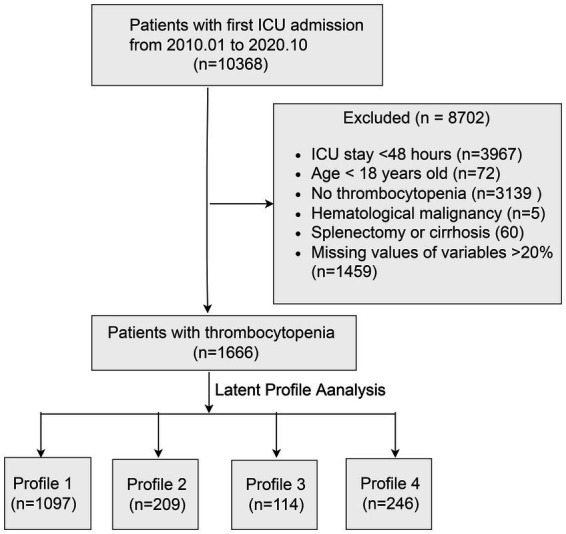
Flow chart of the study. ICU, intensive care unit.

The flow diagram of this study is shown in Figure 1. After excluding 8,702 patients, 1,666 participants with a mean age of 61.5 ± 16.6 years were finally included. Of these, 61.6% were male. The overall mortality rate was 23.4%.

### 3.2. Selection of optimal categories

**Figure 2:**
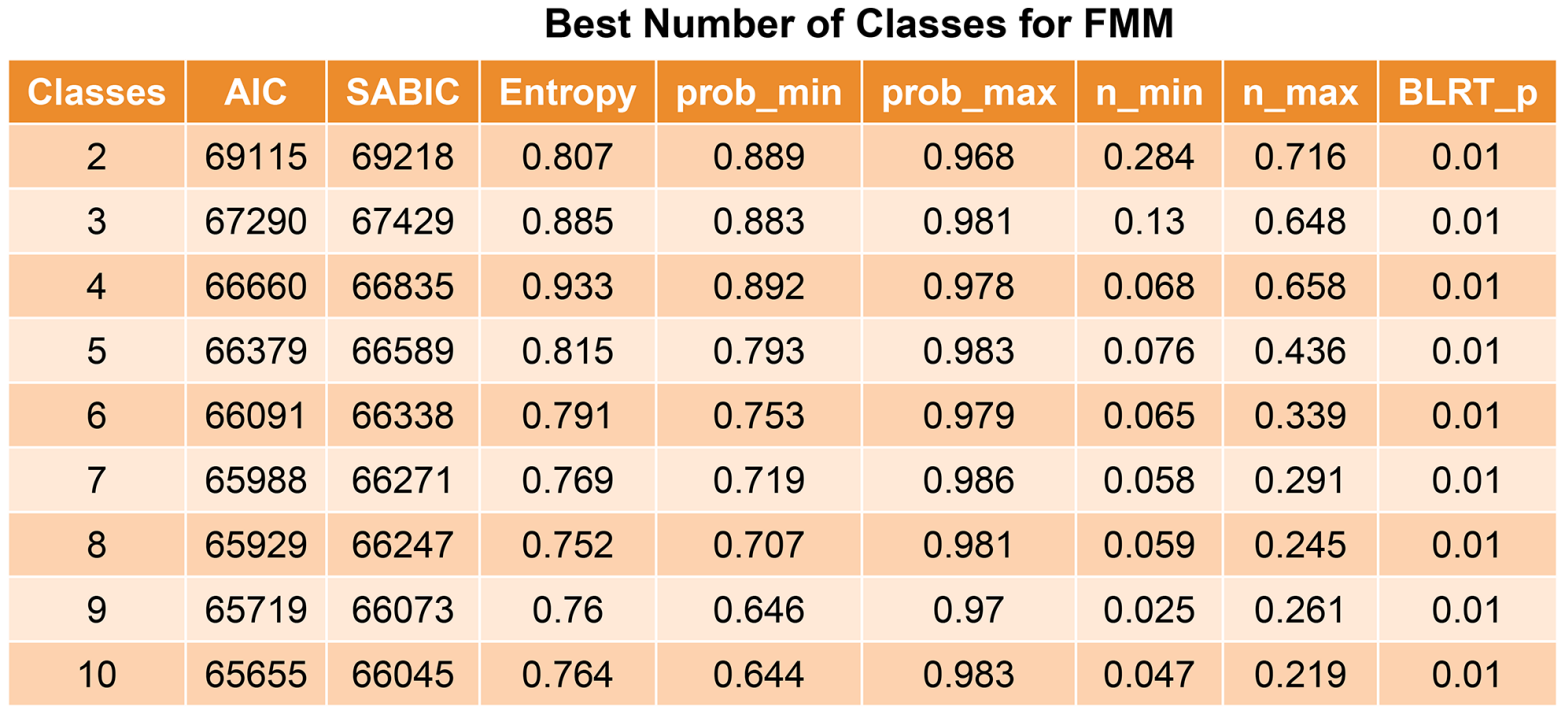
Best number of classes for latent profile analysis. The value of *p* was reported for the bootstrap likelihood ratio test comparing the current model (k class) to the model with k-1 class. AIC, Akaike information criterion; SABIC, sample size-adjusted Bayesian information criteria.

The Akaike information criterion and sample size-adjusted BIC value decreased from the 2-class model to the 10-class model, but the decrease began to slow from the 4-class model to the 5-class model. The 4-class model had the largest entropy and minimum probability of <0.8, starting at the 5-class model, suggesting that the minimum probability assigned to this class was <0.8, and the 5–10-class models were considered unstable (Figure 2). Therefore, the optimal selection was a 4-class model.

### 3.3. Clinical characteristics and outcomes of subphenotypes

**Figure 3:**
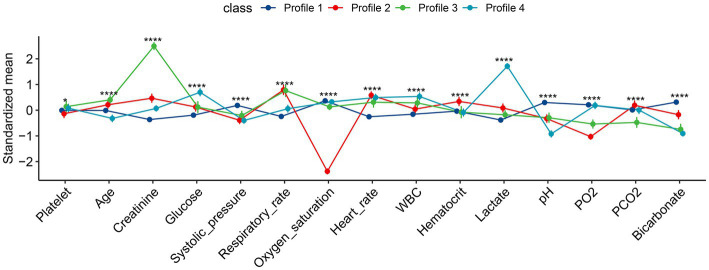
Characteristics of the four subphenotypes identified by latent profile analysis. All numeric values were scaled for better visualization on the vertical axis. Profile 1 is the largest class over all study days with all variables in average value (the baseline class). Profile 2 is characterized by low oxygen saturation and partial pressure of oxygen, the highest partial pressure of carbon dioxide (the respiratory failure class). Profile 3 is characterized by the highest serum creatinine and low bicarbonate levels (renal dysfunction class). Profile 4 is characterized by the highest lactate level, and low systolic pressure and bicarbonate level (the shock class). FMM, finite mixture modeling; WBC, white blood cell; PO_2_, partial pressure of oxygen; PCO_2_, partial pressure of carbon dioxide. *p < 0.05, ****p < 0.001.

The characteristics of the four subphenotypes are shown in Figure 3 and Table 1. Subphenotype 1 was the most abundant one of the four categories, with a total of 1,097 patients, accounting for 66% of all patients. The values of all variables were approximate of the means. Thus, subphenotype 1 was considered as the baseline category. Subphenotype 2 was characterized by low oxygen saturation [94, IQR: 93–96%], low partial pressure of oxygen (97.4 ± 41.9 mmHg), and the highest partial pressure of carbon dioxide (36.1 ± 8.2 mmHg) and was considered as the respiratory failure category. Subphenotype 3 was characterized by the highest serum creatinine level (272, IQR: 216–272 mmol/L) and low bicarbonate levels (17.6 ± 3.7 mmol/L) and was considered as the renal insufficiency category. Subphenotype 4 was characterized by the highest lactate level (7.90, IQR, 6.40–10.05 mmol/L), low systolic blood pressure (116.4 ± 23.8 mmHg), and low bicarbonate level (17.0 ± 2.7 mmol/L) and was considered as the shock category.

**Table 1:** Continuous variables included in the mixture modeling.

Characteristic	Profile 1 (*n* = 1,097)	Profile 2 (*n* = 209)	Profile 3 (*n* = 114)	Profile 4 (*n* = 246)	*p*
Age (years)	61.3 ± 16.6	65.0 ± 15.8	68.2 ± 14.2	56.2 ± 16.5	<0.001
Platelet (×10^9^/L)	87.1 ± 28.7	83.0 ± 33.1	91.7 ± 38.2	89.8 ± 36.8	0.041
White blood cell (×10^9^/L)	11.2 ± 5.0	12.3 ± 6.7	13.7 ± 7.7	15.2 ± 6.1	<0.001
Hematocrit	0.3 ± 0.1	0.3 ± 0.1	0.3 ± 0.1	0.3 ± 0.1	<0.001
pH	7.4 ± 0.1	7.4 ± 0.1	7.4 ± 0.1	7.3 ± 0.1	<0.001
PO2 (mmHg)	171.0 ± 54.9	97.4 ± 41.9	126.6 ± 49.0	169.6 ± 57.8	<0.001
PCO2 (mmHg)	34.8 ± 6.4	36.1 ± 8.2	31.5 ± 7.0	34.8 ± 7.4	<0.001
Bicarbonate (mmol/L)	21.4 ± 2.9	19.7 ± 4.1	17.6 ± 3.7	17.0 ± 2.7	<0.001
Lactate (mmol/L)	2.20 (1.50, 3.20)	2.70 (1.50, 5.00)	2.60 (1.50, 3.68)	7.90 (6.40, 10.05)	<0.001
Creatinine (mmol/L)	70 (55, 91)	106 (70, 170)	272 (216, 272)	90 (71, 121)	<0.001
Glucose (mmol/L)	8.3 (6.8, 10.1)	8.9 (7.2, 11.5)	9.2 (7.2, 11.5)	11.4 (8.9, 13.9)	<0.001
Systolic pressure (mmHg)	133.1 ± 28.8	116.7 ± 26.1	121.9 ± 24.7	116.4 ± 23.8	<0.001
Heart rate (/min)	88.0 ± 19.0	105.4 ± 19.9	99.8 ± 21.2	103.6 ± 20.2	<0.001
Respiratory rate (/min)	14 (12, 16)	18 (14, 26)	18 (14, 25)	14 (12, 19)	<0.001
Oxygen saturation (%)	100 (100, 100)	94 (93, 96)	100 (99, 100)	100 (99, 100)	<0.001

Continuous variables are described by means and quarterbacks. Categories variables are analyzed by *χ*^2^ test and continuous variables are analyzed by Wilcoxon rank sum test. PO2, partial pressure of oxygen; PCO2, partial pressure of carbon dioxide.

**Figure 4:**
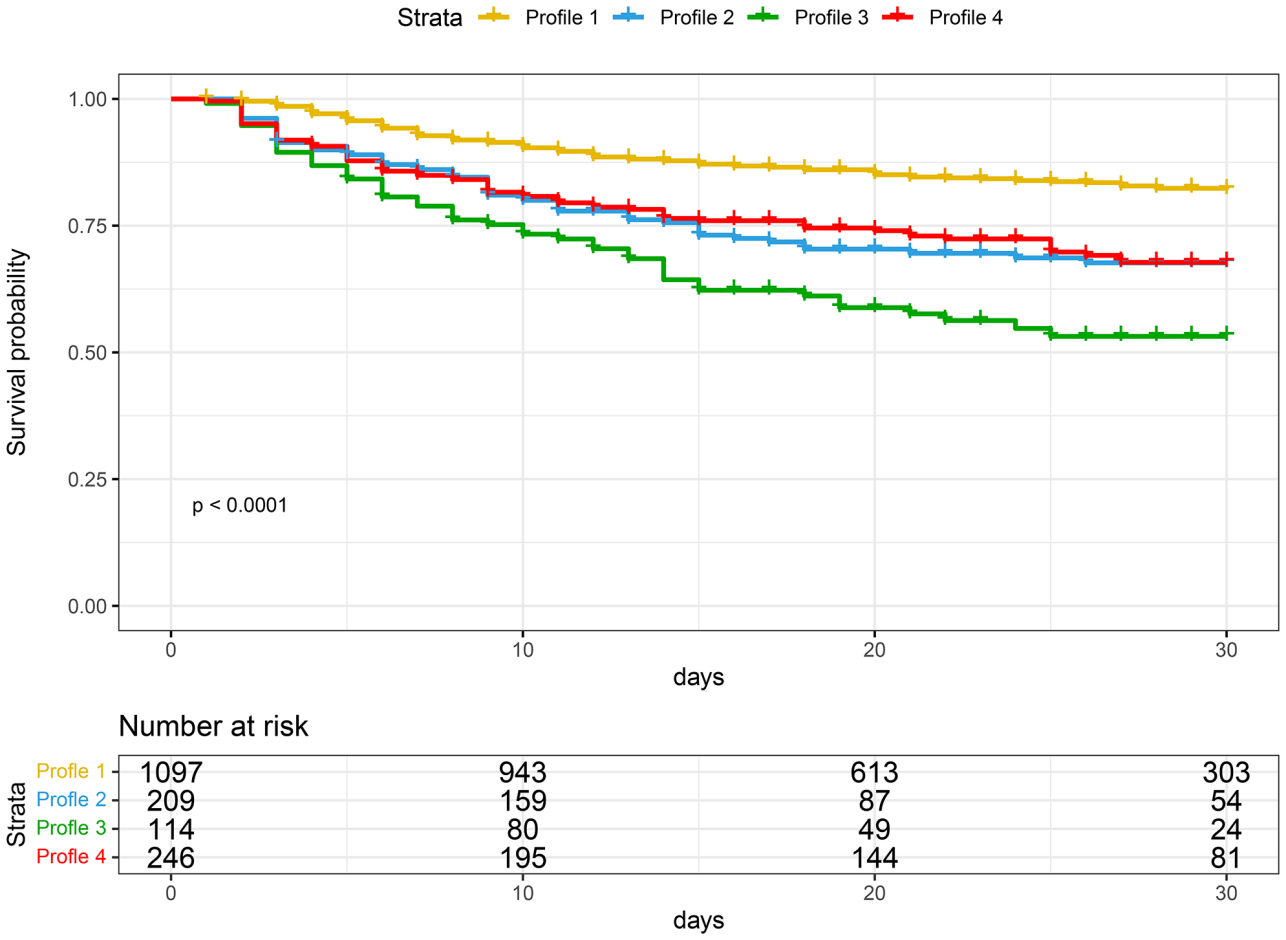
Kaplan–Meier curves for 30-day survival, stratified by four subphenotypes.

Table 2 shows a comparison of clinical outcomes. Subphenotype 1 had the lowest mortality rate (17.4%), the lowest duration of mechanical ventilation, the shortest duration of ICU stay and hospital stay, and the lowest hospitalization cost. Subphenotype 3 had the highest mortality rate (47.4%), the highest APACHE II score (25.0 ± 8.1), and the highest proportion of renal replacement therapy (47.4%). Subphenotype 4 had a mortality rate of 31.3%, the longest duration of hospital stay (23 days, IQR: 12–34 days), and the highest hospital cost (CNY 108 × 10^3^, IQR: CNY 52 × 10^3^–149 × 10^3^). Subphenotypes 2 and 4 had similar mortality rates (Figure 4).

**Table 2:** Categorical variables and outcome variables not included in the mixture modeling.

Characteristic	Profile 1 (*n* = 1,097)	Profile 2 (*n* = 209)	Profile 3 (*n* = 114)	Profile 4 (*n* = 246)	*p*
Male [*n*(%)]	646.0 (58.9%)	143.0 (68.4%)	90.0 (78.9%)	147.0 (59.8%)	<0.001
Smoking [*n*(%)]	377.0 (34.4%)	89.0 (42.6%)	49.0 (43.0%)	90.0 (36.6%)	0.056
Alcohol drinking [*n*(%)]	404.0 (36.8%)	83.0 (39.7%)	48.0 (42.1%)	91.0 (37.0%)	0.637
Comorbidities [*n*(%)]					
Hypertension	312.0 (28.4%)	78.0 (37.3%)	60.0 (52.6%)	58.0 (23.6%)	<0.001
Diabetes	86.0 (7.8%)	28.0 (13.4%)	24.0 (21.1%)	27.0 (11.0%)	<0.001
Congestive heart failure	32.0 (2.9%)	15.0 (7.2%)	13.0 (11.4%)	16.0 (6.5%)	<0.001
Chronic obstructive pulmonary disease	75.0 (6.8%)	35.0 (16.7%)	17.0 (14.9%)	9.0 (3.7%)	<0.001
Input_24h (L/h)	3.91 (3.28, 4.84)	4.02 (3.27, 5.14)	4.27 (2.98, 5.97)	4.66 (3.72, 6.51)	<0.001
Uo_24h (L/h)	2.4 (1.7, 3.1)	2.3 (1.3, 3.2)	1.3 (0.4, 2.3)	2.3 (1.6, 3.1)	<0.001
APACHE-II score	18.2 ± 7.0	21.9 ± 8.3	25.0 ± 8.1	20.8 ± 7.4	<0.001
Vasopressor used [n(%)]	651.0 (59.3%)	167.0 (79.9%)	98.0 (86.0%)	202.0 (82.1%)	<0.001
Glucocorticoid used [n(%)]	460 (41.9)	104 (49.8)	38 (33.3)	123 (50)	0.004
Immunoglobulin used [*n*(%)]	9 (0.8)	13 (6.2)	4 (3.5)	3 (1.2)	<0.001
Platelet infusion [*n*(%)]	130 (11.9)	43 (20.6)	29 (25.4)	59 (24)	<0.001
Renal replacement therapy [*n*(%)]	20.0 (1.8%)	31.0 (14.8%)	54.0 (47.4%)	28.0 (11.4%)	<0.001
Biochemical indexes on ICU admission					
Red blood cell (×10^9^/L)	3.4 ± 0.6	3.6 ± 0.8	3.3 ± 0.8	3.3 ± 0.8	<0.001
Potassium (mmol/L)	4.1 ± 0.5	4.1 ± 0.6	4.5 ± 0.7	4.0 ± 0.6	<0.001
Sodium(mmol/L)	142.0 ± 4.1	142.5 ± 4.6	141.8 ± 5.4	144.5 ± 4.3	<0.001
Calcium (mmol/L)	2.0 ± 0.2	1.9 ± 0.2	1.9 ± 0.2	1.9 ± 0.2	0.013
Urea (mmol/L)	7.3 (5.5, 9.3)	10.3 (7.1, 15.2)	19.6 (14.6, 20.7)	7.9 (6.0, 10.3)	<0.001
Prothrombin time (s)	15.6 (14.5, 16.8)	15.9 (14.4, 18.5)	17.0 (14.9, 19.4)	16.7 (15.0, 20.0)	<0.001
International normalized ratio)	1.25 (1.13, 1.38)	1.28 (1.13, 1.55)	1.38 (1.18, 1.65)	1.35 (1.19, 1.68)	<0.001
Activated partial thromboplastin time (s)	40 (36, 46)	46 (40, 55)	47 (41, 58)	43 (37, 59)	<0.001
D.dimer (μg/L)	5.3 (2.1, 13.6)	7.0 (2.6, 16.0)	6.6 (2.7, 16.0)	8.1 (2.6, 16.0)	0.001
Outcome					
Hospital_mortality [*n*(%)]	191.0 (17.4%)	68.0 (32.5%)	54.0 (47.4%)	77.0 (31.3%)	<0.001
Ventilation duration (days)	2 (1, 7)	4 (1, 9)	4 (0, 10)	3 (1, 10)	0.017
ICU length of stay (days)	5 (3, 11)	7 (4, 13)	7 (3, 14)	7 (4, 13)	<0.001
Length of hospital stay (days)	21 (13, 31)	17 (10, 30)	16 (8, 26)	23 (12, 34)	<0.001
Cost (×10^3^ yuan)	67 (40, 101)	57 (32, 99)	57 (32, 97)	108 (52, 149)	<0.001

Continuous variables are described by means and quarterbacks. Categories variables are analyzed by *χ*^2^ test and continuous variables are analyzed by Wilcoxon rank sum test. APACHE, acute physiology and chronic health evaluation; Input_24h, fluid input for 24 h on ICU admission; Uo_24h, urine volume for 24 h on ICU admission; ICU, intensive care unit; Hosp. LOS, length of hospital stay.

### 3.4. Therapeutic interventions

**Figure 5:**
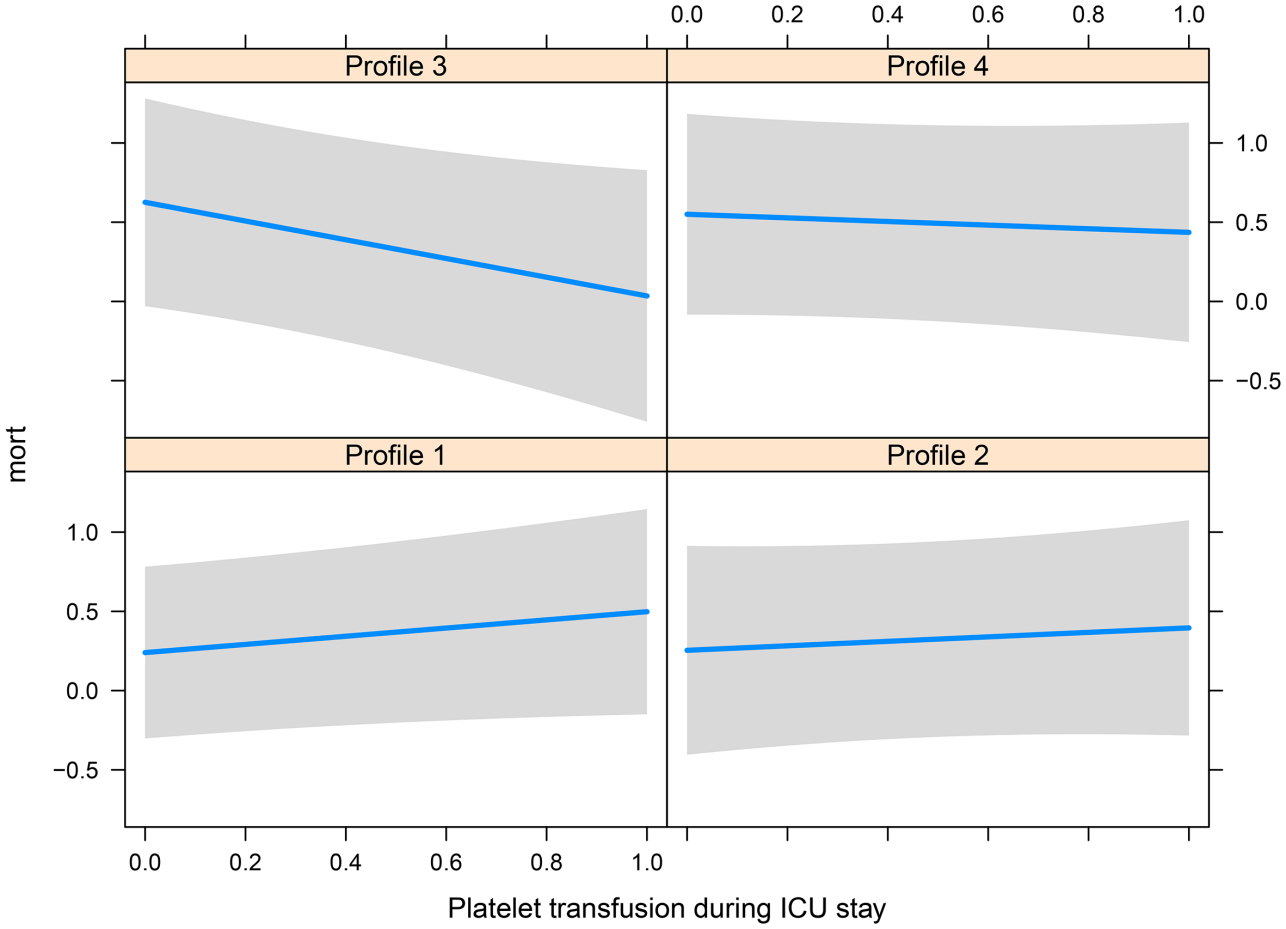
Platelet transfusion and risk of hospital mortality, stratified by four subphenotypes.

**Figure 6:**
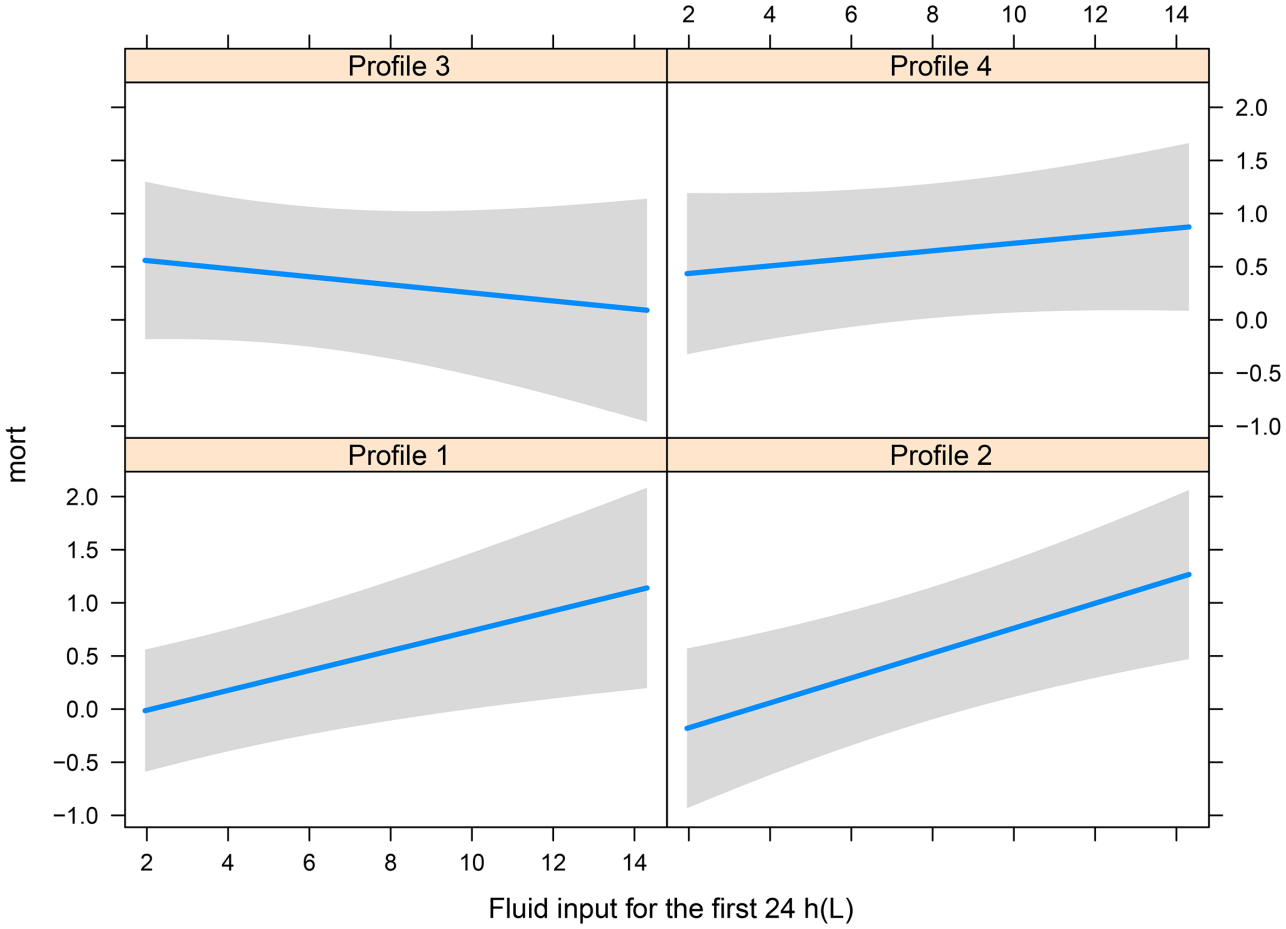
Fluid intake and risk of hospital mortality, stratified by four subphenotypes.

There were significant differences in the proportion of platelet transfusion among the four subphenotypes (*p* < 0.001). Subphenotype 1 had the lowest platelet transfusion rate (11.9%), and others had a platelet transfusion rate of >20%. After adjusting for age, sex, APACHE II score, vasopressor used, white blood cell count, surgery, and sepsis, multivariate Cox regression models indicated that there was a significant interaction between platelet transfusion and each category, with higher platelet transfusion associated with a decreased risk of in-hospital mortality in subphenotype 3 (HR: 0.66, 95% CI: 0.46–0.94; Table 3). The total fluid intake at 24 h after admission to the ICU was 4.0 (IQR: 3.3–5.2) L, and the total urine output was 2.3 (IQR: 1.6–3.1) L in all patients. Subphenotype 1 had the lowest fluid intake (3.91, IQR: 3.28–4.84) L and highest urine output (2.4, IQR: 1.7–3.1) L. Subphenotype 4 had the highest fluid intake (4.66, IQR: 3.72–6.51) L, and subphenotype 3 had the lowest urine output (1.3, IQR: 0.4–2.3) L (Table 2). However, a significant interaction was noted between fluid intake and each category, with higher fluid intake associated with a decreased risk of in-hospital mortality in subphenotype 3 (HR: 0.94, 95% CI: 0.89–0.99 per 1 l increase in fluid intake) but associated with an increased risk of in-hospital mortality in subphenotypes 1 (HR: 1.10, 95% CI: 1.03–1.18 per 1 l increase in fluid intake) and 2 (HR: 1.19, 95% CI: 1.08–1.32 per 1 l increase in fluid intake; Table 4). Figure 5 shows platelet transfusion and risk of hospital mortality, stratified by four subphenotypes, whereas Figure 6 shows fluid intake and risk of hospital mortality, stratified by four subphenotypes.

**Table 3:** Cox’s proportional hazard models for platelet transfusion and hospital mortality in different profiles.

Characteristic	HR	95% CI	*p*
Age	0.84	0.68, 1.03	0.10
Sex	1.14	0.92, 1.42	0.2
APACHE-II score	1.10	1.08, 1.11	<0.001
Vasopressor used	3.43	2.42, 4.84	<0.001
White blood cell	0.97	0.95, 0.99	<0.001
Surgery	0.47	0.38, 0.59	<0.001
Sepsis	0.71	0.56, 0.88	0.002
Class			
Profile 1	—	—	
Profile 2	1.01	0.71, 1.44	0.9
Profile 3	1.48	1.03, 2.13	0.036
Profile 4	1.38	1.00, 1.91	0.053
Interaction between profile and platelet transfusion			
Profile 1	1.17	0.81,1.67	0.4
Profile 2	0.88	0.48, 1.64	0.7
Profile 3	0.66	0.46, 0.94	0.023
Profile 4	0.69	0.39, 1.23	0.2

HR, hazard ratio; CI, confidence interval; APACHE, acute physiology and chronic health evaluation; Input_24h, fluid input for 24 h on ICU admission; ICU, intensive care unit.

**Table 4:** Cox’s proportional hazard models for fluid input and hospital mortality in different profiles.

Characteristic	HR	95% CI	*p*
Age	0.88	0.72, 1.09	0.2
Sex	1.12	0.90, 1.40	0.3
APACHE-II score	1.09	1.08, 1.11	<0.001
Vasopressor used	3.38	2.39, 4.79	<0.001
White blood cell	0.97	0.95, 0.99	<0.001
Surgery	0.47	0.38, 0.59	<0.001
Sepsis	0.72	0.57, 0.90	0.005
Class			
Profile 1	—	—	
Profile 2	0.81	0.44, 1.48	0.5
Profile 3	2.30	1.21, 4.36	0.011
Profile 4	1.76	0.94, 3.28	0.077
Interaction between profile and Input_24h			
Profile 1	1.10	1.03, 1.18	0.005
Profile 2	1.19	1.08, 1.32	0.001
Profile 3	0.94	0.89, 0.99	0.029
Profile 4	1.02	0.93, 1.11	0.7

HR, hazard ratio; CI, confidence interval; APACHE, acute physiology and chronic health evaluation; Input_24h, fluid input for 24 h on ICU admission; ICU, intensive care unit.

### 3.5. Sensitivity analysis

We deleted 203 patients with missing data, retained outliers for sensitivity analysis, and obtained similar results in LPA analysis (Supplementary Figure S1). The maximum value of entropy was in four categories; therefore, the best classification was four categories, and the features of the four categories were also similar.

## 4. Discussion

In this study, we identified four clinical subphenotypes of TP, with different physiological characteristics and in-hospital mortality, using only routine clinical data. We also found an interaction between subphenotypes and platelet transfusion and fluid intake, suggesting the involvement of these subphenotypes in precision medicine-based approaches to the treatment of TP.

Platelet transfusion is a common treatment for patients with PT, but it can be ineffective for various reasons, such as infection, medication, disseminated intravascular coagulation, etc. (24–26). In some cases, platelet counts transiently increase after transfusion, and several studies have demonstrated that platelet transfusion does not improve patient outcomes (27). Our study demonstrated that platelet transfusion can improve in-hospital mortality rates in patients with subphenotype 3 of TP, indicating that identifying subphenotypes is a potential method for addressing platelet transfusion in critically ill patients.

Intravenous fluids are the cornerstone of patient care in the ICU; both inadequate fluid intake and fluid overload increased mortality. Overall, in-hospital mortality increased with higher fluid intake in our study, which is consistent with the finding of previous studies (28, 29). However, in subphenotype 3 cases, increased fluid intake was associated with improved outcomes. This may be associated with the clinical characteristics of subphenotype 3, including renal dysfunction, metabolic acidosis. Most clinicians are now aware that AKI patients require fluid restriction; however, excessive fluid restriction may lead to insufficient effective blood volume (30, 31). Therefore, a more precise volume assessment is necessary for this patient subpopulation. Subphenotype 4 exhibited the highest lactate level, a high fluid intake, and a high urine output but lower mortality than that exhibited by subphenotype 3, which may be related to less fluid overload. Previous studies have demonstrated that fluid overload is positively correlated with mortality in critically ill patients (32, 33). Therefore, we believe that precise fluid management based on subphenotypic classification is a promising future direction.

Previous classifications of TP were based only on the severity of platelet count decrease, and some critically ill patients often presented with transient TP that was not well reflective of patient prognosis or therapeutic efficacy. Wu et al. reviewed three subphenotypes based on possible mechanisms of sepsis-associated TP: increased platelet consumption, decreased platelet production, and increased platelet destruction (34). In a similar study, Bedet et al. used hierarchical clustering of 60 patients with septic shock and identified five subphenotypes of patients with septic TP, which facilitated further understanding of the mechanisms of TP (12). However, their study included 27 endogenous mediators associated with sepsis, and the clinical applicability of this classification system may be limited.

In the present study, the classification of clinical subphenotypes of TP was based on LPA, which can be used to assess continuous indicators commonly measured in clinics. In contrast to cluster analysis, LPA considers measurement errors and uses objective criteria to determine the optimal categories, making it more robust and reliable, with a minimum class membership probability of >0.8 indicating good model stability (35). Similar techniques have been successfully applied to analyze therapeutic heterogeneity among subgroups of ARDS patients (36, 37).

This study had some limitations. First, the nature of the study was retrospective, and no causal inferences could be drawn. Moreover, the variables investigated were selected with reference to previous studies. Information on some underlying variables (such as height and weight) and inflammation-related variables (such as C-reactive protein and procalcitonin levels) was not available. Thus, further validation of our results in prospective studies is required. Second, the study was conducted at a single center and lacked external validation, which may limit the generalizability and reproducibility of the findings. Future research may explore external validation to ensure the robustness and reliability of the subphenotypes identified. Third, while LPA is a useful technique for identifying subgroups within a population, it is still a relatively new and evolving methodology. Further validation and refinement of this technique may be required to ensure its accuracy and reproducibility. Finally, we were unable to exclude patients with specific types of TP, such as TP due to pharmacological factors and immune-related TP. Fortunately, the overall proportion of such cases was small and did not affect the final results.

## 5. Conclusion

We identified four subphenotypes of patients with TP in the ICU, with different prognoses and different responses to therapeutic interventions, using common biochemical indicators and vital signs. These findings can improve our understanding of the heterogeneity of patients with TP and can be used as a basis for future studies. In addition, these findings may facilitate the identification of different subphenotypes of TP for better individualized treatment of patients in the ICU.

## Data availability statement

The raw data supporting the conclusions of this article will be made available by the authors, without undue reservation.

## Ethics statement

The studies involving human participants were reviewed and approved by DRY-2023-YX-016. Written informed consent for participation was not required for this study in accordance with the national legislation and the institutional requirements.

## Author contributions

WZ and XJ carried out the design. XJ analyzed the data and drafted the manuscript. YP revised the manuscript. XC supervised the study. All authors contributed to the article and approved the submitted version.

## Funding

This study was supported by the Conba Hospital Management project of the Zhejiang Hospital association (grant number 2021ZHA-KEB335) and the Dongyang Science and Technology Bureau (grant number 21-337).

## Conflict of interest

The authors declare that the research was conducted in the absence of any commercial or financial relationships that could be construed as a potential conflict of interest.

## Publisher’s note

All claims expressed in this article are solely those of the authors and do not necessarily represent those of their affiliated organizations, or those of the publisher, the editors and the reviewers. Any product that may be evaluated in this article, or claim that may be made by its manufacturer, is not guaranteed or endorsed by the publisher.

## Footnotes

^1^ https://www.r-project.org/

## Abbreviations

AKI: Acute kidney injury
APACHE: Acute physiology and chronic health evaluation
ARDS: Acute respiratory distress syndrome
BIC: Bayesian information criterion
CI: Confidence interval
ICU: Intensive care unit
LPA: Latent profile analysis
LRT: Likelihood ratio test
HR: Hazard ratio
TP: Thrombocytopenia
