# Supplementary material for: Identification of subphenotypes in critically ill thrombocytopenic patients with different responses to therapeutic interventions: a retrospective study
Source: Front Med (Lausanne). 2023 Apr 27;10:1166896. doi: 10.3389/fmed.2023.1166896 (PMC10174319; doi:10.3389/fmed.2023.1166896)
Supplement: Supplementary file 1 [file Data_Sheet_1.PDF]

Table 1: STROBE Statement—checklist of items that should be included in reports of observational studies.

	Item No.	Recommendation	Page No.	Relevant text from manuscript
**Title and abstract**	1	(*a*) Indicate the study's design with a commonly used term in the title or the abstract	1	Title
		(*b*) Provide in the abstract an informative and balanced summary of what was done and what was found	1	Abstract
**Introduction**				
Background/rationale	2	Explain the scientific background and rationale for the investigation being reported	2	Introduction
Objectives	3	State specific objectives, including any prespecified hypotheses	2	Introduction
**Methods**				
Study design	4	Present key elements of study design early in the paper	3	Study Design
Setting	5	Describe the setting, locations, and relevant dates, including periods of recruitment, exposure, follow-up, and data collection	3	Study Design and Data collection
Participants	6	(*a*) *Cohort study*—Give the eligibility criteria, and the sources and methods of selection of participants. Describe methods of follow-up<br>*Case-control study*—Give the eligibility criteria, and the sources and methods of case ascertainment and control selection. Give the rationale for the choice of cases and controls<br>*Cross-sectional study*—Give the eligibility criteria, and the sources and methods of selection of participants	3	Study Design and Data collection
		(*b*) *Cohort study*—For matched studies, give matching criteria and number of exposed and unexposed<br>*Case-control study*—For matched studies, give matching criteria and the number of controls per case		
Variables	7	Clearly define all outcomes, exposures, predictors, potential confounders, and effect modifiers. Give diagnostic criteria, if applicable	3	Data collection and Diagnostic criteria

Data sources/ measurement	8*	For each variable of interest, give sources of data and details of methods of assessment (measurement). Describe comparability of assessment methods if there is more than one group	3	Data collection
Bias	9	Describe any efforts to address potential sources of bias	4	Statistical Analyses
Study size	10	Explain how the study size was arrived at	NA	
Quantitative variables	11	Explain how quantitative variables were handled in the analyses. If applicable, describe which groupings were chosen and why	4	Statistical Analyses
Statistical methods	12	(*a*) Describe all statistical methods, including those used to control for confounding	4	Statistical Analyses
		(*b*) Describe any methods used to examine subgroups and interactions	4	Statistical Analyses
		(*c*) Explain how missing data were addressed	3	Data Processing
		(*d*) *Cohort study*—If applicable, explain how loss to follow-up was addressed<br>*Case-control study*—If applicable, explain how matching of cases and controls was addressed<br>*Cross-sectional study*—If applicable, describe analytical methods taking account of sampling strategy	NA	
		(*e*) Describe any sensitivity analyses	4	Statistical Analyses
**Results**				
Participants	13*	(a) Report numbers of individuals at each stage of study—eg numbers potentially eligible, examined for eligibility, confirmed eligible, included in the study, completing follow-up, and analysed	4	Study population
		(b) Give reasons for non-participation at each stage	4	Study population
		(c) Consider use of a flow diagram		Figure 1
Descriptive data	14*	(a) Give characteristics of study participants (eg demographic, clinical, social) and information on exposures and potential confounders		Table 1-2
		(b) Indicate number of participants with missing data for each variable of interest	NA	
		(c) *Cohort study*—Summarise follow-up time (eg, average and total amount)	NA	
Outcome data	15*	*Cohort study*—Report numbers of outcome events or summary measures over time		Table 2
		Case-control study—Report numbers in each exposure category, or summary measures of exposure		
		Cross-sectional study—Report numbers of outcome events or summary		

		measures		
Main results	16	(*a*) Give unadjusted estimates and, if applicable, confounder-adjusted estimates and their precision (eg, 95% confidence interval). Make clear which confounders were adjusted for and why they were included	5-6	Results, Table 3-4
		(*b*) Report category boundaries when continuous variables were categorized		
		(*c*) If relevant, consider translating estimates of relative risk into absolute risk for a meaningful time period		
Other analyses	17	Report other analyses done—eg analyses of subgroups and interactions, and sensitivity analyses		Table 3-4
**Discussion**				
Key results	18	Summarise key results with reference to study objectives	6	Discussion
Limitations	19	Discuss limitations of the study, taking into account sources of potential bias or imprecision. Discuss both direction and magnitude of any potential bias	7	Discussion
Interpretation	20	Give a cautious overall interpretation of results considering objectives, limitations, multiplicity of analyses, results from similar studies, and other relevant evidence	6	Discussion
Generalisability	21	Discuss the generalisability (external validity) of the study results	6	Discussion
**Other information**				
Funding	22	Give the source of funding and the role of the funders for the present study and, if applicable, for the original study on which the present article is based	8	Funding

*Give information separately for cases and controls in case-control studies and, if applicable, for exposed and unexposed groups in cohort and cross-sectional studies.

Note: An Explanation and Elaboration article discusses each checklist item and gives methodological background and published examples of transparent reporting. The STROBE checklist is best used in conjunction with this article (freely available on the Web sites of PLoS Medicine at http://www.plosmedicine.org/, Annals of Internal Medicine at http://www.annals.org/, and Epidemiology at http://www.epidem.com/). Information on the STROBE Initiative is available at www.strobe-statement.org.

Figure S1. Best number of classes for latent profile analysis

Best Number of Classes for FMM

Classes	AIC	SABIC	Entropy	prob_min	prob_max	n_min	n_max	BLRT_p
2	134535	134631	0.888	0.886	0.986	0.157	0.843	0.01
3	133606	133734	0.925	0.888	0.982	0.094	0.761	0.01
4	132265	132427	0.955	0.899	0.982	0.043	0.742	0.01
5	131901	132096	0.836	0.81	0.977	0.035	0.495	0.01
6	131756	131984	0.822	0.759	0.969	0.035	0.463	0.01
7	131021	131283	0.881	0.681	0.999	0.011	0.544	0.01
8	130983	131278	0.837	0.721	0.99	0.011	0.434	0.01
9	130787	131115	0.848	0.742	0.997	0.011	0.453	0.01
10	130708	131069	0.794	0.635	0.998	0.008	0.347	0.01

The p-value was reported for the bootstrap likelihood ratio test comparing the current model (k class) to the model with k-1 class.
Abbreviations: AIC, Akaike information criterion; SABIC, sample size-adjusted Bayesian information criteria
